# Guideline Adherence for Intrapartum Group B Streptococci Prophylaxis in Penicillin-Allergic Patients

**DOI:** 10.1155/2013/917304

**Published:** 2013-02-12

**Authors:** Kimberly A. Paccione, Harold C. Wiesenfeld

**Affiliations:** Department of Obstetrics, Gynecology, and Reproductive Sciences, Magee-Womens Hospital, University of Pittsburgh School of Medicine, Pittsburgh, PA 15213, USA

## Abstract

*Objective*. To investigate adherence to the 2002 Centers for Disease Control and Prevention (CDC) guidelines for perinatal group B streptococci (GBS) prevention in penicillin-allergic obstetric patients. *Methods*. This is a retrospective cohort study of penicillin-allergic obstetric patients who tested positive for GBS and delivered at our institution in 2010. Electronic medical records were reviewed for the nature of the penicillin allergy, documentation of having previously tolerated cephalosporins, gestational age at delivery, type of delivery, antimicrobial sensitivity testing, and antibiotics administered. Antimicrobial sensitivity testing and “appropriate” antibiotic choice, which was determined using 2002 CDC guidelines, were analyzed. *Results*. Intrapartum antibiotic prophylaxis was administered in 97.8% (95% confidence interval [CI] 93.5–99.5%) of patients, but it was considered appropriate in only 62.2% (95% CI 53.8–70.0%) of patients. Clindamycin was the most commonly used antibiotic, but 26.4% (95% CI 16.3–39.7%) of patients who received clindamycin did not have confirmation of susceptibility via antimicrobial sensitivity testing. Overall, the sensitivity testing was performed in only 65.5% (95% CI 56.2–73.7%) of patients in whom it was indicated. *Conclusion*. Compliance with CDC guidelines for performing antimicrobial sensitivity testing and choosing an appropriate antibiotic in GBS-positive penicillin-allergic women continues to be suboptimal. Institution of measures to increase adherence is necessary.

## 1. Introduction

Group B streptococci (GBS) is the most frequent bacterial pathogen in neonates and is the leading cause of early-onset sepsis and meningitis in the USA [1]. The single most important risk factor for early-onset GBS infection is maternal colonization [1, 2]. The universal screening for maternal GBS colonization at 35 to 37 weeks' gestation and the use of intrapartum antibiotic prophylaxis have resulted in a nearly 80% reduction in the rate of neonatal GBS infection over the past 15 years, from 1.7 cases per 1,000 live births in the early 1990s to 0.34–0.37 cases per 1,000 live births in recent years [1].

 The recommended antibiotic for GBS prophylaxis is penicillin. However, at least 10% of patients report an allergy to penicillin [3, 4]. Prior to 2002, the antibiotics of choice for GBS prophylaxis in penicillin-allergic obstetric patients were clindamycin or erythromycin [5]. However, the emergence of resistance to these antibiotics among GBS isolates resulted in revision of perinatal GBS prevention guidelines from the Centers for Disease Control and Prevention (CDC) in 2002 [6, 7]. These guidelines recommend that a history of the patient's penicillin allergy be assessed and that women determined to be at low risk of anaphylaxis receive intrapartum cefazolin. For all other GBS-positive, penicillin-allergic patients, testing of the GBS isolate for susceptibility to erythromycin and clindamycin is recommended. If the isolate is resistant to clindamycin or erythromycin or the susceptibility is unknown, then vancomycin should be used. 

 Despite the existing national recommendations outlining the management of GBS colonization of the penicillin-allergic obstetric patient, suboptimal adherence to the guidelines has been reported, potentially leaving many neonates exposed to GBS [1, 8, 9]. We studied adherence to CDC guidelines for intrapartum GBS prophylaxis in penicillin-allergic patients, determining the rate of antimicrobial sensitivity testing and the rate of selection of appropriate antibiotic prophylaxis.

## 2. Materials and Methods

We performed a retrospective cohort study of GBS-positive obstetric patients with a reported penicillin allergy who delivered at Magee-Womens Hospital of UPMC in 2010. Nearly 10,000 deliveries occur each year at this academic medical center in Pittsburgh, PA, USA. Approval was obtained from UPMC's Quality Improvement Review Board (QI no. 0000665) prior to initiating the study.

**Figure 1:**
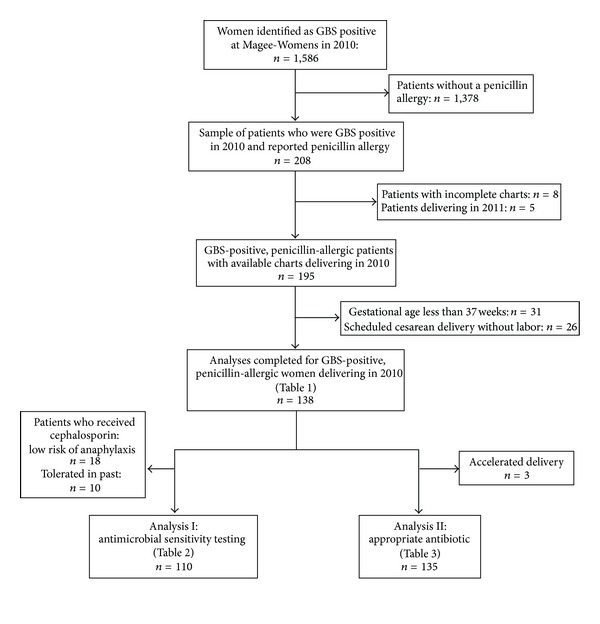
Flow Chart of Patient Selection.

 The patient sample for this study was identified using a computer-generated list of all obstetric patients who tested positive for rectovaginal GBS in 2010. A total of 1,586 GBS-positive obstetric patients were identified. We individually examined each GBS-positive patient's inpatient and outpatient electronic medical records for report of penicillin allergy prior to delivery. Of the 1,586 GBS-positive patients, 208 had a recorded penicillin allergy (13.1%).  Figure 1 shows a flow chart of patient selection in this study.

 Data were abstracted from the electronic medical records onto a standardized data collection spreadsheet. Abstracted data included age, gravidity, parity, race/ethnicity, type of insurance, type of obstetric provider, gestational age at delivery, type of delivery, recorded nature of the penicillin allergy, other pertinent allergies, documentation of having tolerated a cephalosporin in the past, antimicrobial sensitivity testing, and antibiotics received. We excluded 5 patients who delivered after 2010 and 8 patients whose electronic medical records contained incomplete information. We also excluded patients who delivered before 37 weeks of gestation (*n* = 31) because their GBS culture results might not be available at the time of delivery as well as patients who had a scheduled cesarean delivery without labor (*n* = 26) because GBS prophylaxis is not recommended in this population.

 We performed two analyses of the sample of GBS-positive, penicillin-allergic patients delivering at Magee-Womens Hospital in 2010: (1) comparison of patients whose GBS isolates underwent antimicrobial sensitivity testing with those whose GBS isolates did not, and (2) comparison of patients who received appropriate antibiotic with those who did not. Antimicrobial sensitivity testing was considered “performed” if it was documented in the patient's medical record or in our laboratory database. “Appropriate antibiotic” was defined according to 2002 CDC guidelines [7]. Cephalosporin was defined as appropriate for patients at low risk of anaphylaxis (those who had no immediate hypersensitivity reaction to penicillin or had previously tolerated a cephalosporin). Clindamycin or erythromycin was considered appropriate if the GBS isolate was susceptible to both of these antibiotics. Vancomycin was deemed an appropriate choice if the patient was at high or unknown risk of anaphylaxis and antimicrobial sensitivity was unknown or the GBS isolate was resistant to either clindamycin or erythromycin.

 Statistical analyses included the Wilcoxon rank-sum test to compare medians, Fisher's exact test to compare categorical variables, and the Agresti-Coull method (aka the modified Wald method) to calculate 95% confidence intervals (CI) for binomial proportions.

## 3. Results 

In 2010, 1,586 obstetric patients tested positive for rectovaginal GBS, and the prevalence of self-reported penicillin allergy was 13.1% (95% CI 11.5–14.9%). 

 After applying our exclusion criteria, the final sample size was 138 patients. Characteristics of all patients eligible for at least one of the analyses are described in Table 1. The nature of the patient's penicillin allergy was documented in 78 out of 138 (56.5%) women. Overall, 135 out of 138 (97.8%) patients received antibiotic prophylaxis at the time of labor. The majority of these patients received clindamycin (40.0%).

 In our first analysis, we compared GBS-positive, penicillin-allergic women who did and did not receive antimicrobial sensitivity testing. Sensitivity testing was deemed unnecessary in 28 patients who were determined to be at low risk of anaphylaxis. Specimens from the remaining 110 women should have undergone antimicrobial sensitivity testing (Table 2). Antimicrobial sensitivity testing was performed in 65.5% (95% CI 56.2–73.7%) of these patients. There was no difference between women whose GBS isolates underwent sensitivity testing and those whose GBS isolates did not in terms of age, gravidity, parity, race/ethnicity, type of insurance, and type of obstetric provider. Clindamycin was the most commonly used antibiotic, but 26.4% (95% CI 16.3–39.7%) of patients who received clindamycin did not have antibiotic sensitivity testing performed on their GBS isolates.

 In our second analysis, we compared GBS-positive, penicillin-allergic women who did and did not receive an appropriate antibiotic. Three patients who experienced a rapid delivery did not receive antibiotic prophylaxis. Of the remaining 135 women, we found that 84 (62.2%, [95% CI 53.8–70.0%]) received an appropriate antibiotic (Table 3). There was no difference between women who received an appropriate antibiotic and those who did not in terms of age, gravidity, parity, race/ethnicity, type of insurance, and type of obstetric provider. Twenty-eight out of 135 (20.7%) patients received clindamycin or erythromycin without confirmation of susceptibility of their GBS isolates. Seven out of 135 (5.2%) patients received vancomycin inappropriately when they could have received clindamycin or erythromycin based on antibiotic sensitivity testing or a cephalosporin due to low risk of anaphylaxis. Fourteen women (10.4%) inappropriately received cephalosporin despite having a history of a type I hypersensitivity reaction to penicillin without documentation of having previously tolerated cephalosporins.

## 4. Discussion and Conclusion

Although substantial progress has been made in the prevention of early-onset GBS disease, GBS continues to be the leading infectious cause of morbidity and mortality among newborns in the USA [1]. The CDC revised its perinatal GBS prevention guidelines in 2002 due to emergence of resistance to clindamycin and erythromycin among GBS isolates in the USA and Canada (7–25% for erythromycin and 3–15% for clindamycin). By following these guidelines, most cases of early-onset GBS disease can be prevented. The 2002 CDC guidelines were implemented as per our hospital's official guidelines immediately after they were published, and all providers were made aware of the updates at that time. In our study, despite the high rate of intrapartum antibiotic administration for GBS-positive, penicillin-allergic obstetric patients (97.8%), CDC guidelines for obtaining antimicrobial sensitivity testing and choosing an appropriate antibiotic were not consistently followed.

 One of the most significant actions that providers can take to increase effective GBS prophylaxis is obtaining a more detailed clinical history of the patient's penicillin allergy. At our institution, the nature of the penicillin allergy was documented in only 56.5% of patients. In many circumstances, a more detailed reaction history would have avoided the need for antimicrobial sensitivity testing. Many patients who report a penicillin allergy are not actually allergic to penicillin but instead have experienced side effects of the antibiotic. Additionally, many patients have had reactions to penicillin that are not type I hypersensitivity reactions or have tolerated other beta-lactam agents in the past, putting them at low risk of anaphylaxis to cephalosporins. A recent literature review of the use of cephalosporins in penicillin-allergic patients estimates cross-reactivity to be approximately 1% with first-generation cephalosporins (e.g., cefazolin) [10]. Anaphylactic reactions to cephalosporins are rare, with the overall incidence estimated to be between 0.0001 and 0.1% [11]. Therefore, a patient whose reaction history is not suggestive of a type I hypersensitivity reaction to penicillin may reasonably be prescribed a cephalosporin for GBS prophylaxis.

 Excluding those patients eligible for cephalosporin therapy due to a low risk of anaphylaxis, all other penicillin-allergic patients should have antimicrobial sensitivity testing performed on their GBS isolates. At our institution, however, 34.5% of women whose isolates should have undergone antimicrobial sensitivity testing did not receive such testing. Of these women, over one-third (36.8%) received clindamycin without confirmation of susceptibility. With only 48% of GBS isolates at our hospital demonstrating susceptibility to clindamycin, many infants potentially could have been exposed to GBS. All patients with isolates that had unknown sensitivities should have instead received vancomycin because of concern for resistance.

 Based on the 2002 CDC guidelines, 62.2% of our patient sample received an appropriate antibiotic. Of those women who did not receive appropriate antibiotic prophylaxis, 55% received either clindamycin or erythromycin when antibiotic sensitivity testing either was not performed or revealed resistance to clindamycin or erythromycin. In these circumstances, patients may have received inadequate prophylaxis, placing their neonates at increased risk for GBS disease. Administration of cephalosporins occurred in 27.5% of patients receiving inappropriate prophylaxis. These patients had a recorded history of a type I hypersensitivity reaction to penicillin without documentation of having previously tolerated cephalosporins, placing them at increased risk of anaphylaxis. Of the remaining patients who received an inappropriate antibiotic, 14% received vancomycin when they could have received clindamycin or erythromycin based on antibiotic sensitivity testing or cephalosporin due to low risk of anaphylaxis. While vancomycin is a highly effective prophylactic treatment, its use is generally restricted because of emerging vancomycin resistance among some gram-positive organisms. Inappropriate administration of vancomycin could result in selection of resistance in other organisms.

 This study had several strengths. First, the electronic medical records allowed us to identify eligible patients by generating a complete list of all GBS-positive obstetric patients delivering at Magee-Womens Hospital in 2010. We used this list to establish our study sample by thoroughly reviewing each patient's medical record to determine if a penicillin allergy had been reported. Second, our ability to view data from both inpatient and outpatient data sources as well as our laboratory database allowed accurate and complete data collection. Only 8 charts out of 203 had incomplete information that prevented us from including these patients.

 One limitation of our study is that patients who had GBS testing performed at other institutions would not have been captured in the initial list of patients who tested positive for rectovaginal GBS at Magee-Womens Hospital. Therefore, some patients who could have been eligible for the study might have been missed. Also, because this study is retrospective, it is limited by the information that was recorded in the patients' charts. It is possible that providers obtained more information than was documented and used that information (e.g., type of allergic reaction to penicillin) in their decision-making. A related limitation is that penicillin allergy was defined by patient report, not by objective methods such as skin testing for allergic sensitivity. A final limitation of the study is that new CDC guidelines for intrapartum GBS prophylaxis were released in November of 2010, which could have affected the behavior of providers during the last two months of the collected data. These new guidelines, however, had similar recommendations for antibiotic sensitivity testing and appropriate antibiotic choice [1]. One change in the recommendations is that erythromycin is no longer considered as an appropriate alternative for GBS prophylaxis, regardless of sensitivity testing results. However, only two patients received erythromycin in our cohort, and each administration was inconsistent with both the 2002 and the 2010 guidelines. Therefore, the results of our study were not affected. 

 Universal screening and the use of intrapartum antibiotic prophylaxis have resulted in a significant reduction in the rate of neonatal GBS infection. The annual incidence of early-onset GBS disease was reduced by 33% in the three years after the 2002 revised guidelines were issued [12]. Although significant progress has been made, adherence to CDC guidelines for GBS prophylaxis in penicillin-allergic patients continues to be challenging. While providers at our institution were made aware of the CDC guidelines when they were initially published, interventions including ongoing educational programs for obstetric providers and prompts in the laboratory ordering system and electronic medical records may help to improve compliance with GBS prophylaxis guidelines. As a result of the findings of this study, renewed efforts to improve guideline adherence at our hospital have evolved, focusing on the provider education regarding the 2010 CDC guidelines as well as the development of specific institutional protocols to encourage the acquisition of penicillin reaction histories when screening for GBS positivity and to facilitate antimicrobial sensitivity testing and appropriate antibiotic selection. Because of the severe consequences that could arise from suboptimal compliance with GBS prophylaxis guidelines, other institutions should consider investigating their own adherence. By optimizing guideline adherence in the penicillin-allergic obstetric population, further reduction in the incidence of early-onset GBS disease can be achieved. 

## Figures and Tables

**Table 1:** Characteristics of the GBS+, penicillin-allergic patients who delivered at Magee-Womens hospital in 2010 (postexclusion criteria).

Characteristics	Number	Percentage (95% confidence interval [CI])
Total *n*	138	
Median age (y)	30 (16–41)	
Median gravidity	2 (1–9)	
Median parity	1 (0–5)	
Race/ethnicity		
Non-Hispanic White	114	82.6 (75.4–88.1)
Non-Hispanic African-American	23	16.7 (11.3–13.8)
Other	1	0.7 (0–4.4)
Type of insurance		
Private	97	70.3 (62.2–77.3)
Medical assistance	41	29.7 (22.7–37.8)
Obstetrical provider Type		
Private physician	100	72.5 (64.5–79.3)
Maternal fetal medicine	3	2.2 (0.5–6.5)
OB/GYN residents	25	18.1 (12.5–25.4)
Midwife	8	5.8 (2.8–11.2)
Family practitioner	2	1.4 (0.07–5.5)
Type of delivery		
Vaginal	116	84.1 (77.0–89.3)
Cesarean after labor	22	15.9 (10.7–23.0)
Patient documentation of nature of the penicillin allergy	78	56.5 (48.2–64.5)
Patients with documentation that they received antibiotic prophylaxis	135	97.8 (93.5–99.5)
Type of antibiotics received		
Cephalosporin	41	30.4 (23.2–38.6)
Clindamycin	54	40 (32.1–48.4)
Erythromycin	2	1.5 (0.07–5.6)
Vancomycin	36	26.7 (19.9–34.7)
Penicillin	2	1.5 (0.07–5.6)

**Table 2:** GBS+, penicillin-allergic patients who delivered at Magee-Womens Hospital in 2010. Comparisons between women who had antibiotic sensitivity testing and women who did not.

Characteristics	Underwent sensitivity testing		Did not undergo sensitivity testing		*P *
	Number	Percent (95% CI)	Number	Percent (95% CI)
Total (*n* = 110)	72		38		
Race/Ethnicity					0.79
Non-Hispanic White	60	83.3 (72.9–90.4)	31	81.6 (66.3–91.1)	
Non-Hispanic African-American	11	15.3 (8.6–25.5)	7	18.4 (8.9–33.7)	
Other	1	1.4 (0–8.2)	0	0.0 (0–10.9)	
Type of insurance					0.05
Private	54	75 (63.8–83.6)	21	55.3 (39.7–69.9)	
Medical assistance	18	25 (16.4–36.2)	17	44.7 (30.1–60.3)	
Obstetric provider type					0.23
Private physician	54	75 (63.8–83.6)	28	73.7 (57.8–85.2)	
Maternal fetal medicine	3	4.2 (0.9–12.0)	0	0.0 (0–10.9)	
OB/GYN residents	12	16.7 (9.6–27.1)	7	18.4 (8.9–33.7)	
Midwife	3	4.2 (0.9–12.0)	1	2.6 (0–14.7)	
Family practitioner	0	0.0 (0–6.1)	2	5.3 (0.5–18.2)	
Type of antibiotics received					0.019
Cephalosporin	7	9.7 (4.5–19.0)	7	18.4 (8.9–33.7)	
Clindamycin	39	54.2 (42.7–65.2)	14	36.8 (23.3–52.8)	
Erythromycin	2	2.8 (0.2–10.2)	0	0.0 (0–10.9)	
Vancomycin	24	33.3 (23.5–44.9)	12	31.6 (19.0–47.6)	
Penicillin	0	0.0 (0–6.1)	2	5.3 (0.5–18.2)	
None	0	0.0 (0–6.1)	3	7.9 (2.0–21.5)	

**Table 3:** GBS+, penicillin-allergic patients who delivered at Magee-Womens Hospital in 2010. Comparisons between women who received appropriate antibiotic* and women who did not.

Characteristics	Received appropriate antibiotic		Did not receive appropriate antibiotic		*P *
	Number	Percent (95% CI)	Number	Percent (95% CI)
Total (*n* = 135)	84		51		
Race/ethnicity					0.47
Non-Hispanic White	72	85.7 (76.5–91.8)	41	80.4 (67.4–89.2)	
Non-Hispanic African-American	12	14.3 (8.2–23.5)	9	17.6 (9.3–30.5)	
Other	0	0.0 (0–5.2)	1	2 (0–11.3)	
Type of insurance					0.05
Private	65	77.4 (67.3–85.1)	31	60.8 (47.1–73.0)	
Medical assistance	19	22.6 (14.9–32.7)	20	39.2 (27.0–52.9)	
Obstetric provider type					0.7
Private physician	61	72.6 (62.2–81.1)	37	72.5 (59.0–83.0)	
Maternal fetal medicine	2	2.4 (0.2–8.8)	1	2 (0–11.3)	
OB/GYN residents	14	16.7 (10.1–26.2)	11	21.6 (12.3–34.8)	
Midwife	6	7.1 (3.0–15.0)	1	2 (0–11.3)	
Family practitioner	1	1.2 (0–7.1)	1	2 (0–11.3)	
Type of antibiotics received					0.006
Cephalosporin	27	32.1 (23.1–42.8)	14	27.5 (17.0–41.0)	
Clindamycin	28	33.3 (24.2–44)	26	51 (37.7–64.1)	
Erythromycin	0	0.0 (0–5.2)	2	3.9 (0.3–14.0)	
Vancomycin	29	34.5 (25.2–45.2)	7	13.7 (6.5–26.0)	
Penicillin	0	0.0 (0–5.2)	2	3.9 (0.3–14.0)	

*Appropriate antibiotic is defined according to 2002 CDC guidelines.

(i) Cephalosporin for patients at low risk of anaphylaxis (those who had no immediate hypersensitivity reaction to penicillin or had previously tolerated a cephalosporin).

(ii) Clindamycin or erythromycin for patients whose GBS isolates were susceptible to both of these antibiotics.

(iii) Vancomycin for patients at high or unknown risk of anaphylaxis when antimicrobial sensitivity was unknown or the GBS isolates were resistant to either clindamycin or erythromycin.
